# Development differentially sculpts receptive fields across early and high-level human visual cortex

**DOI:** 10.1038/s41467-018-03166-3

**Published:** 2018-02-23

**Authors:** Jesse Gomez, Vaidehi Natu, Brianna Jeska, Michael Barnett, Kalanit Grill-Spector

**Affiliations:** 1 Neurosciences Program, Stanford University School of Medicine, Stanford, CA 94305 USA; 2 Psychology Department, Stanford University, Stanford, CA 94305 USA; 3 Stanford Neurosciences Institute, Stanford University, Stanford, CA 94305 USA

## Abstract

Receptive fields (RFs) processing information in restricted parts of the visual field are a key property of visual system neurons. However, how RFs develop in humans is unknown. Using fMRI and population receptive field (pRF) modeling in children and adults, we determine where and how pRFs develop across the ventral visual stream. Here we report that pRF properties in visual field maps, from the first visual area, V1, through the first ventro-occipital area, VO1, are adult-like by age 5. However, pRF properties in face-selective and character-selective regions develop into adulthood, increasing the foveal coverage bias for faces in the right hemisphere and words in the left hemisphere. Eye-tracking indicates that pRF changes are related to changing fixation patterns on words and faces across development. These findings suggest a link between face and word viewing behavior and the differential development of pRFs across visual cortex, potentially due to competition on foveal coverage.

## Abstract (web summary)

Population receptive fields (pRFs) in the visual system are key information-processors, but how they develop is unknown. Here, authors use fMRI and pRF modeling in children and adults to show that in the ventral stream only pRFs in face- and word-selective regions continue to develop, mirroring changes in viewing behavior.

## Introduction

The receptive field (RF), the portion of visual space from which information is processed, is a fundamental characteristic of the visual system. RFs are found from the earliest stages of the visual system in retinal ganglion neurons^1^, to the first visual field area, V1^2^, to high-level visual regions^3–6^ including regions involved in face^4,5^ and word processing^6^. Given behavioral differences across children and adults in both low-level (e.g., visual acuity^7^) and high-level (e.g. face recognition^8^) visual behaviors reliant on RFs, it is possible that RFs continue to develop across the entire ventral stream after age 5. However, several fundamental questions remain unanswered: (1) Do RFs in human visual cortex develop during childhood? (2) If so, what is the nature of the development? (3) What is the relationship between RF development and viewing experience? Understanding RF development will provide fundamental insight into the most basic computation underlying the function of over 30% of the human brain. With disorders such as dyslexia and autism having been associated with atypical brain processing as well as uncharacteristic fixations patterns^9,10^, understanding the link between RF development and viewing experience has broad implications in neuroscience.

High-level visual abilities such as reading and face recognition rely on a series of visual computations across the ventral visual stream^11^: a hierarchy of visual areas beginning with V1 and culminating in ventral temporal cortex (VTC) where face^12^ and word-selective^13^ regions supporting face^14^ and word-form perception^15^, respectively, are located. Since neurons across the entire ventral visual hierarchy have RFs^3–6,16^ and neurons with similar RFs are spatially clustered^2^, the population receptive field (pRF) of neurons in each fMRI voxel can be reliably measured^17^. In each of early (V1–V3) and intermediate visual areas (V4–VO1) in the ventral stream, pRFs systematically tile the visual field and are organized topographically across the cortical surface into visual field maps^16,17^. In contrast, in high-level ventral regions that are involved in reading^6^ and face recognition^4,5^, pRFs are large and always cover the central visual field. Consequently, the visual field coverage (VFC) of face and character-selective regions is non-uniform and concentrated around the center of the visual field— referred to as a foveal bias^18^.

The wiring of the visual system, which determines neurons’ RFs and topographic organization, is laid out during embryonic development by molecules that guide axon generation and synaptic formation^19–21^. While recent data suggest that the hierarchical, topographic organization of visual cortex is present at birth^22^, prevailing thought is that molecular cues alone cannot specify the precision of adult RFs and visual field maps^19,21^. It is thus unknown if development of RFs occurs early after birth or is protracted across childhood. Thus, we asked: if and how do pRFs in the ventral stream develop during childhood? We considered three possibilities: (i) pRF development occurs during early infancy, predicting that pRFs, VFC, and visual field maps across the entire visual stream are adult-like by age 5. (ii) There is a gradient of development, whereby earlier visual areas develop prior to higher-level regions in the ventral stream. This hypothesis predicts that pRFs and VFC in early visual areas are adult-like by age 5 even as pRFs and VFC in high-level category-selective regions continue to develop past age 5. This hypothesis is based on empirical findings showing that functional^23–26^ and anatomical^27,28^ development of face and character-selective regions is protracted compared to earlier regions^29^. (iii) pRFs and VFC across the entire ventral stream continue to develop past age 5 into adulthood.

In light of the systematic link between the cortical representations of face/character-selective regions and foveal representations^18,30,31^ it is particularly interesting to consider the predictions of developmental hypotheses in high-level visual cortex. One prediction is that pRFs and eccentricity representations in VTC develop early^22^ (before age 5), with face and character-selectivity emerging on top of regions containing adult-like pRFs and foveal bias. Another prediction is that a rough foveal preference in the lateral VTC initially biases the emergent position of face/word-regions, but that viewing experience associated with increasingly foveal fixations will lead to prolonged development of pRFs and foveal bias in these regions. Researchers^30,31^ have also hypothesized that competition between representations of faces and words on foveal resources during development, together with left lateralization of the language system in the brain, is what generates the adult left brain lateralization for words and right lateralization for faces^30,31^. This hypothesis predicts differential development of pRFs and VFC in face and character-selective regions across hemispheres. For this study, we use the term character-selective to refer to word-selective regions. Lastly, pRF development may be related to viewing behavior, as it has been shown that adults tend to fixate on the center of faces^18,32^ (nose bridge) putting informative features^33,34^ at the region with the highest acuity. However, it is unknown if children fixate on faces and words in the same way as adults, in which case viewing experience shapes pRFs, or if their viewing patterns develop together with the development of pRFs in a developmental interplay.

To elucidate the development of pRFs and visual field maps in the ventral visual stream, we modeled pRFs with functional magnetic resonance imaging (fMRI, see Methods section) in children (*n* = 26, 5 to 12 years old) and adults (*n* = 26, 22–27 years old). Participants were scanned as they viewed a sweeping checkerboard bar while fixating on a central stimulus and performing a color-change task on the fixation. We modeled the pRF of each voxel in the ventral stream as a 2-dimensional Gaussian with a nonlinearity, referred to as compressive spatial summation (CSS)^4,35^. CSS improves pRF fits in higher-level visual areas^4,35^.

We examined: (i) if there are qualitative differences across age-groups in pRF properties and visual field maps, (ii) if there are quantitative differences across age-groups in pRF size, pRF eccentricity, and VFC obtained by the collection of pRFs spanning each visual area, and (iii) if developmental effects differ across regions constituting the ventral visual stream. Then, we examined if there is a relationship between pRF development and viewing behavior. A subset of participants participated in a behavioral experiment outside the scanner on a different day in which they freely viewed images of faces and words during a recognition task while their fixations were eye tracked. We tested if fixation patterns on faces and words differed between children and adults and if so, whether they were related to pRF properties measured separately during fMRI.

Our data reveal differential development of visual regions constituting the ventral visual stream, whereby early stages (retinotopic areas V1 through VO1) demonstrate no qualitative or quantitative differences across age-groups in pRF size, pRF eccentricity, or VFC obtained by the collection of pRFs spanning each visual area. However, higher-level regions selective for faces and characters differentially develop across hemispheres. Developmental changes are most striking near the center of the visual field (fovea), with face-selective cortex in the right hemisphere and character-selective cortex in the left hemisphere gaining privileged coverage of the central visual field by adulthood. Notably, these developments have behavioral consequences. We find that not only do children’s natural viewing of faces and pseudowords differ from adults, but children’s fixations mirror the coverage of the visual field in right face-selective and left character-selective regions measured separately with fMRI.

## Results

### Early and intermediate visual areas are developed by age 5

All participants completed pRF mapping. There were no significant differences across age-groups in (i) motion during fMRI (adult motion average: 0.7 ± 0.33 mm, child: 0.89 ± 0.2 mm; *t*(39) = 1.4, n.s.), (ii) fixation behavior during fMRI (*t*(30) = 1.73, n.s. Supplementary Fig. 1A, B), or (iii) task performance during fMRI (*t*(14) = 1.28, n.s., Supplementary Fig. 1C). To test the goodness-of-fit of the pRF model, we measured the mean variance explained by the model for V1 voxels in each participant and compared across age-groups. We matched groups on the variance explained by the pRF model in V1 voxels by excluding eight children with the lowest V1 model fits and three adults with the highest V1 model fits. This matching resulted in no significant differences across groups in the percentage variance explained by the pRF model across visual regions (Supplementary Fig. 2C). These quality assurance metrics ensure that any developmental effects are not due to differences between age-groups in motion, performance during fMRI, pRF model fits, or measurement noise.

**Fig. 1:**
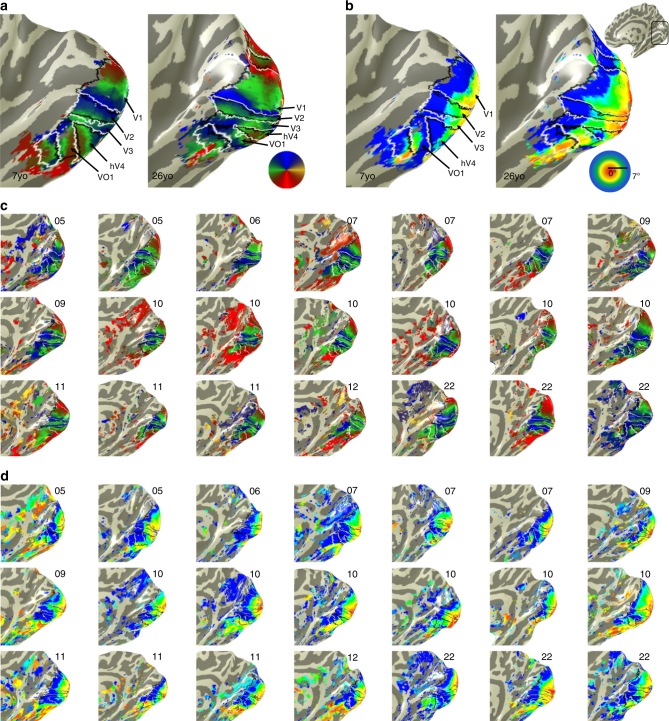
Early visual areas show no qualitative development. **a** Polar angle maps from an example 7-year-old and 26-year-old. Color wheel: polar angle. Data are shown within an anatomical mask encompassing the central 7°. **b** Eccentricity maps in the same participants. Color wheel: eccentricity. Inset brain shows zoomed region in black outline. **c** Polar angle maps and **d** eccentricity maps in the right hemisphere of all child participants and three example adults. Numbers indicate the age of the participant. Maps are of the central 7° and are thresholded at 5% variance explained, voxel level. Lines: boundaries of visual field maps. Polar angle and eccentricity maps of all participants and both hemispheres are shown in Supplementary Figures 3–6

Examination of the topographic organization of polar angle and eccentricity maps revealed that these maps were qualitatively similar across age-groups (Fig. 1, Supplementary Figs. 3–6, all participants’ maps). That is, children, like adults, displayed a series of mirror-reversed polar angle maps (Fig. 1a, c) emerging from a hemi-field representation in and around the calcarine sulcus (corresponding to V1, see Methods section for map definitions) and two sets of large-scale eccentricity maps, one spanning the occipital cortex, in which eccentricities progressively increase from posterior to anterior, and one in VTC, in which eccentricities progressively increase from lateral to medial (Fig. 1b, d).

Using polar angle and eccentricity maps, we successfully defined visual areas V1 through VO1 bilaterally in all 18 children and all 23 adults (Fig. 1, Supplementary Figs. 3–6). The cortical volume of visual field maps was slightly (<5%) smaller in children than adults (Supplementary Fig. 2A), but like adults, over 90% of voxels were driven by the mapping stimulus and could be modeled by a pRF (Supplementary Fig. 2B).

**Fig. 2:**
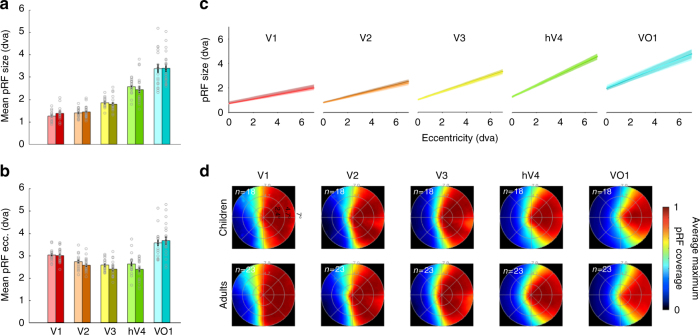
pRF properties in V1–VO1 are quantitatively similar across development. **a** Mean pRF size in V1–VO1 across 18 children (light colors) and 23 adults (dark colors); Error bars: standard error. Gray circles: individual participant data. Each circle is a participant. **b** Mean pRF eccentricity in V1–VO1 of the same participants. Gray circles: individual participant data. **c** pRF size vs. eccentricity relationship is similar across age-groups. The line of best fit (solid line) and the standard error (shaded region) illustrates the relationship between pRF eccentricity and size in units of degrees of visual angle (dva). Adults are shown in dark colors (*n* = 23), children (*n* = 18) in light colors (each age group is shown in a separate graph in Supplementary Fig. 7). Fits are calculated in each participant, slopes and intercepts are then averaged across participants. **d** Visual field coverage of V1–VO1 computed using the average maximum pRF density coverage for each participant and then averaged across participants. Maps are averaged across hemispheres by flipping the right hemisphere data. Top: children. Bottom: adults. Number of participants is indicated in the top-left of each panel. Inner to outermost ring segments correspond to 2.4, 4.7, and 7 degrees of visual angle (dva)

Notably, there were no significant differences across age-groups in mean pRF size (Fig. 2a) or mean pRF eccentricity in V1–VO1 (Fig 2b) (2-way analysis of variance (ANOVA), with factors of ROI and age-group, no main effects of age: Fs(1,195) < 1.1, ps > 0.36). Further, in V1–VO1, there was no correlation between mean pRF size and age (−0.11 < Rs(41) < 0.12, n.s.) or mean eccentricity and age (−0.04 < Rs(41) < 0.29, n.s.). In children’s V1–VO1, like in adults’, pRF size linearly increased with eccentricity (Fig. 2c). Likewise, there were no significant differences across children and adults in either the slopes (*F*_(1,195)_ = 0.39, n.s., 2-way ANOVA with factors of visual area and age group) or intercepts (*F*_(1,195)_ = 2.98, n.s., 2-way ANOVA) of the pRF size vs. eccentricity line fits in V1–VO1.

In children, like adults, pRF size also increased across the visual hierarchy, demonstrated by the progressive steepening of slopes of the size vs. eccentricity line from V1 to VO1 (Fig. 2c, Supplementary Fig 7) and the systematic increase in mean pRF size ascending the visual hierarchy (Fig. 2a, main effect of visual area on pRF size: *F*_(1,195)_ = 188.8, *p* < 0.001, 2-way ANOVA with factors of age and area).

As there were no quantitative differences in pRF properties across children and adults in V1–VO1, the VFC obtained by the collection of pRFs spanning each of these visual field maps was strikingly similar across children and adults (Fig. 2d). In each of V1 through VO1, the VFC was largely uniform and spanned a hemi-field in each hemisphere in both children and adults. There was no significant difference in the total VFC of V1 through VO1 across development (main effect of age group: *F*_(1,244)_ = 1.76, n.s., 2-way ANOVA with factors of age-group and ROI).

To validate that the lack of differences between age-groups does not stem from using the same data to define area boundaries and pRF properties, we repeated analyses using an independently defined V1. That is, we projected the average V1 from the FreeSurfer average brain to each of our participants' brains and repeated the analyses in this independently defined V1. Results showed no differences in pRF size, pRF eccentricity, or VFC, verifying our results (Supplementary Fig. 8). Together, these analyses reveal that past the age of 5, children have adult-like polar angle and eccentricity maps, and adult-like pRF properties and VFC in V1–VO1.

### Coverage of face-selective and character-selective regions develops

**Fig. 3:**
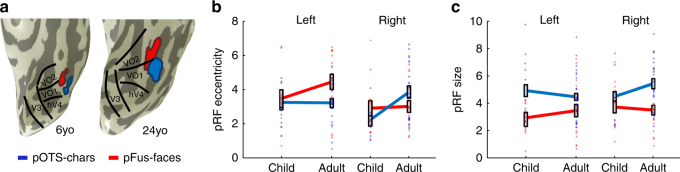
Development of pRF eccentricity and size in face and word regions.** a** pFus-faces (red) and pOTS-chars (blue) on the left ventral temporal lobe in representative child and adult participants. Black lines: boundaries of visual field maps. **b** Mean pRF eccentricity in pFus-faces (red) and pOTS-chars (blue) in children and adults, units are degrees of visual angle. Children are in light colors. **c** Mean pRF size in pFus-faces (red) and pOTS-chars (blue) in children and adults, units are degrees of visual angle. **b**, **c** Error bars: standard error across participants of an age group. Colored circles: individual participant data. Left pOTS-chars: 12 children, 22 adults; Right pOTS-chars: 8 children, 21 adults. Left pFus-faces: 11 children, 18 adults; Right pFus-faces 14 children, 18 adults

To examine if pRFs in high-level regions develop with age, we next defined face-selective and character-selective (i.e., word-selective) regions in all participants using an independent localizer (Fig. 3a, see Methods section). Then we compared across age-groups mean pRF size, eccentricity, and the VFC of each of these regions. Example ROIs shown in Supplementary Fig. 9. We focus on a face-selective region on the posterior fusiform gyrus (pFus-faces) and a character-selective region in the posterior occipitotemporal sulcus (pOTS-chars; Methods) because (i) these regions are proximal to the VO1/VO2 transition in VTC, and (ii) a substantial number of voxels in these regions were modulated by the checkerboard mapping stimulus and therefore could be fit by the pRF model (Supplementary Fig. 2E–F). It is noteworthy that in face-selective pFus-faces and character-selective pOTS-chars children had (i) significantly more voxels that were modulated by the pRF mapping stimulus than adults (Supplementary Fig 2E, *F*_(1,115)_ = 5.68, *p* < 0.02, 2-way ANOVA with factors of ROI and age) and (ii) significantly higher percentage variance explained by the pRF model compared to adults (Supplementary Fig 2F,* F*_(1,114)_ = 8.24, *p* < 0.005, 2-way ANOVA with factors of ROI and age). In general, the size of these regions was not significantly different across age-groups (Supplementary Fig 2D,* F*_(1,115)_ = 0.44, n.s., 2-way ANOVA with factors of ROI and age), except that pFus-faces was numerically larger in adults than children. This difference in voxel number is smaller considering children had more voxels driven by the bar stimulus in face-selective regions than adults. Additionally, in these regions, there was no correlation between mean pRF size and age or mean eccentricity and age either when considering all participants or just children (0.35 > Rs > −0.24, n.s.), justifying the grouping of children into one group.

Different from preceding visual field maps, we found development of pRF properties in pFus-faces and pOTS-chars that varied across hemispheres and regions (Fig. 3b). A 3-way ANOVA on pRF eccentricity with factors of age, hemisphere, and ROI revealed a significant three-way interaction (*F*_(1,111)_ = 4.33; *p* = 0.03) and a significant effect of age (*F*_(1,111)_ = 4.83, *p* = 0.03). Specifically, pRF centers become significantly more eccentric in the right hemisphere for pOTS-chars (*t*(26) = 2.4, *p* = 0.02, post hoc *t*-test) and trended to be more eccentric in the left hemisphere for pFus-faces (*t*(26) = 1.4, *p* = 0.17, post hoc *t*-test, Fig. 3b). A separate 3-way ANOVA on pRF size with the same factors revealed a significant effect of ROI (*F*_(1,111)_ = 13.99, *p* = 0.0003), with pRFs sizes in pOTS-chars about 56% larger than in pFus-faces, and a trending but non-significant three-way interaction (*p* = 0.1; Fig. 3c).

**Fig. 4:**
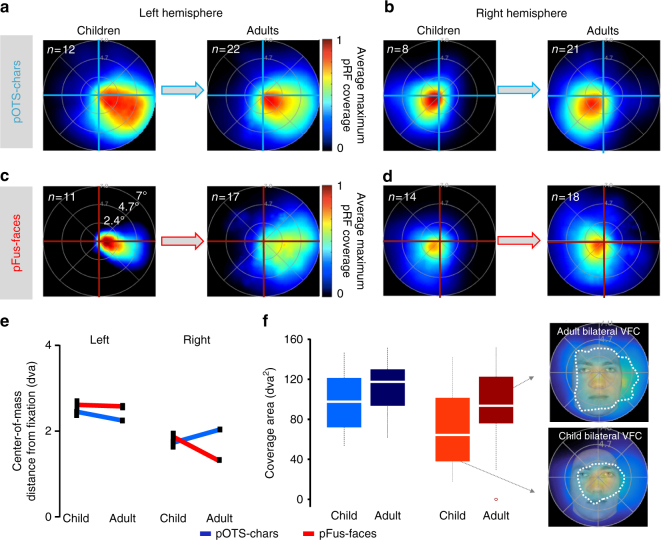
Hemispheric asymmetry in coverage emerges in face and word regions. **a**–**d** For each region, visual field coverage (VFC) was calculated using the average maximum pRF density coverage for each participant and then averaged across participants. The number of participants used to produce the VFC is indicated in the upper left of each panel. Innermost to outermost rings correspond to 2.4, 4.7, and 7 degrees of visual angle (dva), respectively. Arrows illustrates development from childhood to adulthood. **a** VFC of left pOTS-face. **b** VFC of right pOTS-chars. **c** VFC of left pFus-faces. **d** VFC of right pFus-faces. **e** Center-of-mass (CoM) distance of the VFC from fixation illustrated for left and right pOTS-chars and pFus-faces across children and adults. A CoM distance of 2 indicates that the VFC is 2 degrees of visual angle from fixation. Error bars: jackknife standard errors. **f** Left: VFC of bilateral pFus-faces and pOTS-chars in children and adults; white: indicating the median; box: 25th and 75th percentiles; whiskers: range. Right: overlay of the VFC of bilateral pFus-faces in adults (top) and children (bottom) on a face about 1 m from the observer (corresponding to ~6.5 dva). Dashed white contour: 50% density contour of the VFC. This contour covers more of the average-sized face in typical viewing distance in adults than children

As the VFC obtained by the collection of pRFs spanning a region depends on the distribution of pRF sizes and eccentricities as well as their scatter (see Supplementary Fig. 10), subtle development in mean properties may have a profound effect on VFC in face-selective and character-selective regions. To examine this possibility, we estimated the VFC of face-selective and character-selective regions in each participant, separately for each hemisphere, and then measured the mean VFC of these regions across participants of an age group. As in V1–VO1 there were qualitative similarities in the VFC of face-selective and character-selective regions across age-groups. In both children and adults, the VFC of these regions exhibited a contralateral preference, a foveal bias, and a greater coverage of the lower than upper visual field (Fig. 4a–d), as reported previously in adults^4,6^. That is, in each hemisphere, pRFs of face and character-selective regions covered more prominently the contralateral and central visual field than the ipsilateral or peripheral visual field.

pFus-faces and pOTS-chars, however, differ in their developmental patterns across hemispheres. Specifically, we find significant changes in the VFC spanned by pRFs of pFus-faces and pOTS-chars across children and adults (2-dimensional Komolgorov-Smirnov (K-S) test comparing the VFC of children and adults: left pOTS-chars K-S = 0.31, *p* < 0.01; right pOTS-chars K-S = 0.25, *p* < 0.01; right pFus-faces K-S = 0.13, *p* < 0.01; left pFus-faces K-S = 0.32, *p* < 0.01).

To further describe these developmental changes in the VFC, we computed the center-of-mass of the VFC (CoM, reflecting how far the center of the VFC is from fixation, see Methods) in each region and age-group. In the left hemisphere, the CoM shifts towards the fovea across development in left pOTS-chars (Fig. 4a, e**-**left), becoming in adulthood closer to the fovea compared to neighboring left pFus-faces (Fig. 4c, e-left). In the right hemisphere, developmental changes in VFC are reversed: right pOTS-chars pRFs shift away from the fovea (Fig. 4c, e-right), while in right pFus-faces the CoM moves towards the fovea (Fig. 4d, e-right). This interaction between ROI, hemisphere, and age-group was significant (*F*_(1,115)_ = 148, *p* < 0.001, 3-way ANOVA). The CoM of right pFus-faces and left pOTS-chars was significantly more foveal in adults compared to children (ts > 4.13, ps < 0.001).

Despite no significant difference in ROI size between groups (Supplementary Fig. 2D), adult pFus-faces is ~30% larger than children. To test if ROI size influences results, we dilated children’s pFus-faces to match the mean adult size, and repeated analyses. Results remain the same (Supplementary Fig 11), verifying that between-group differences stem from pRF development.

In addition to developmental changes in the CoM, we find significant developmental increases in the total extent of VFC across both hemispheres. That is, the total area of the VFC spanned by pRFs across bilateral pFus-faces and bilateral pOTS-chars significantly increases by ~25 square degrees of visual angle from childhood to adulthood (main effect of age, ANOVA, *F*_(1,93)_ = 6.27, *p* < 0.02, Fig. 4f). Together, these data reveal differential development of the VFC in face-selective and character-selective regions across hemispheres, and an increase in the total extent of VFC.

### Viewing patterns mirror pRF changes in high-level regions

Previous work suggests that optimal viewing behavior involves central fixations, as the center of the stimulus is the most informative region for recognition of faces and words. This framework predicts similar fixation in children and adults. However, development of the VFC in face-selective and character-selective regions suggests that this neural development may impact viewing behavior on faces and words, respectively. We hypothesized that if VFC by pRFs guides natural viewing behavior, the optimal behavior would be to place the VFC, not the fovea, onto the center of stimuli. For children, this predicts fixations that are biased off of the center, resulting in systematic shifts in the viewing of faces and words across children and adults. Specifically, the neural data make three predictions: (i) due to the larger foveal bias in adults, they will show more central fixations than children, (ii) if the VFC in right pFus-faces guides fixation on faces, children’s fixations on faces will be more rightward and upward biased than adults, and (iii) if left pOTS-words drives fixations on words, children’s fixations on words will be more leftward and upward biased than adults.

We assessed natural viewing of faces and words in a subset of our participants (12 children and 11 adults) in a separate behavioral experiment. Outside the scanner, each participant first viewed a series of images from different categories (including faces and pseudowords) and performed a one-back task. Then, participants completed a surprise, self-paced old-new recognition task during which their eye movements were recorded (see Methods section). We then determined if free-viewing fixation patterns followed the predictions of the VFCs obtained from the fMRI experiment inside the scanner where participants were fixating.

**Fig. 5:**
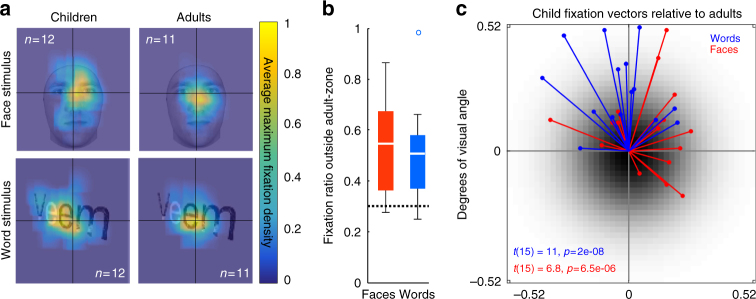
Biases in fixation patterns on faces and words mirror pRF development. **a** Average maximum fixation density maps produced from 12 children and 11 adults during a free-viewing recognition task are overlaid on an example face (top row) and pseudoword (bottom row) stimulus. Fixation density patterns in adults are more centrally placed on faces and words than in children. **b** The mean ratio of fixations in children made outside of an adult-like zone defined as the region where 70% of adults or greater fixated on the stimulus. The dashed line at 0.3 denotes the expected adult value. White: median; box: 25th and 75th percentiles; whiskers: range; circles: outliers; C: children; A: adults; red: unfamiliar faces; blue: pseudowords. **c** Vectors describing the bias in child fixation densities for all face (red) and pseudoword (blue) stimuli relative to the center of adult fixation densities for each stimulus. Each vector is the bias for a particular stimulus. Black Gaussian center represents the centrally-biased adult fixation densities. *t*-tests evaluate if the angular distance of vectors from the null quadrant are significantly different from zero. The null quadrant is defined as the lower left quadrant for faces and lower right for words (see Methods)

Results show that fixation locations on face and pseudoword stimuli differed between children and adults. As shown for two example stimuli, adults foveate more centrally within face and pseudoword stimuli, while children’s fixations are more eccentric across the stimulus expanse (Fig. 5a). Across all images, average fixation patterns were significantly different between children and adults for both face and word stimuli (2D K-S test, ps < 0.001). To further quantify differences in fixation patterns across age-groups, we measured the region of the image in which adults make most of their fixations by calculating for each face and pseudoword stimulus the central region in which adults made 70% of their fixations. Then, we calculated for each child and each image the proportion of fixations made outside of the adult fixation zone (AFZ) and then derived the mean proportion of such fixations across child participants. Results indicate that children fixate significantly outside of the central AFZ for both face (*t*(11) = 4, *p* < 0.01) and word (*t*(11) = 3.63, *p* < 0.01) stimuli (Fig. 5b, tests are relative to the 30% chance level), whereby about 50% of their fixations are outside the adult central fixation zone, even as they make fewer fixations than adults (Supplementary Fig. 12).

Critically, it is not the case that children make more variable fixations than adults, as they show systematic biases in their fixation patterns. Notably, these biases mirror the asymmetries in VFC of face-selective and character-selective regions in their dominant hemispheres. As shown for the example stimuli, children tend to bias their fixations towards the upper right side of faces (Fig. 5a) which puts the VFC of right pFus-faces, which is biased to the left and lower visual field, in a location where it optimally covers the face. Similarly, children tend to fixate on the leftward aspect of words (Fig. 5a), putting the VFC of the left pOTS-chars, which covers the right horizontal visual field, in a place where it optimally covers the word. We quantified this fixation bias by calculating the center-of-mass of fixation densities on each face and pseudoword stimulus separately for adults and children. In Fig. 5c, we plot for each image the vector representing the displacement of child fixation densities relative to adults. Strikingly, children are significantly biased to the upper right quadrant for faces (*t*(15) = 6.8, *p* < 0.001) and the upper left quadrant for words (*t*(15) = 11, *p* < 0.001, see Methods). Importantly, there is no stimulus on which children fixate into the visual field quadrant containing their pRFs (lower left for faces, lower right for words), which would move their VFC further from the stimulus. Results replicate when we exclude the initial 10% of the viewing duration for each stimulus (Supplemental Fig. 12C). Together, behavioral measurements during natural viewing strikingly show that both adults and children fixate in a manner that puts their VFC in face-selective and word-selective regions on the informative region of the visual stimulus.

## Discussion

Modeling population RFs in human visual cortex for the first time in children, we find evidence for differential trajectories of development within the ventral stream and across hemispheres. Early and intermediate visual areas V1–VO1 are developed early, while high-level visual regions in VTC show protracted development in representation of the fovea and VFC from childhood to adulthood. Importantly, fixation patterns on face and pseudoword stimuli during natural viewing demonstrate a link between viewing behavior and developmental changes in the VFC by pRFs in face-selective and character-selective regions. These data provide insight into the possible role of visual experience in sculpting the spatial window through which high-level visual regions process visual information.

We find no qualitative or quantitative difference in visual field topography or pRFs in early and intermediate visual areas V1–VO1 across children and adults. These data suggest that RF properties and visual field maps in the human ventral stream are developed by age 5, consistent with predictions of developmental theories based on research in animal V1^19–21^ and retinotopic mapping in V1–V3^29^.

May this lack of difference across age groups be due to methodological issues? We consider three potential measurement concerns: (1) Using the same data to estimate pRF properties and boundaries of retinotopic areas, as is standard in the field^16,17^, (2) masking of between-group differences due to pRF variability across individuals^36,37^, and (3) obscuring of hV4 by the venous eclipse. First, while it is preferential to use independent data to define an ROI and extract its voxels’ properties, our methodological choice only biases pRFs on the boundaries, which are a small proportion of the ROI, and cannot determine pRF distributions, size vs. eccentricity relationships, or the overall VFC. Further, results replicate in an independently and anatomically defined V1 (Supplementary Fig. 8). Future research could use different data for defining ROIs and estimating pRF properties, which can be accomplished with collecting additional data and/or using standardized atlases, which yet need to be developed for pediatric data. Second, the variance in our data is small (Fig. 2), which suggests that the lack of observed developmental differences in early and intermediate retinotopic areas is likely not due to between-subject variability. Third, differences in measurement artifacts across age groups such as the venous eclipse would have made child and adult hV4 data appear different, which is opposite from what we find (Fig. 2). Together, we believe that the lack of pRF development observed in V1–VO1 is not due to methodological issues. Future longitudinal studies of children may have higher sensitivity to detect more subtle development of pRFs and probe additional pRF features not examined here, including surround suppression^38^, CSS^35^, sensitivity to spatial and temporal frequency^39,40^, and attentional effects^4,41^.

In contrast to early visual field maps, we find developmental changes in pRF centers and VFC in face-selective and character-selective regions in the same participants. Thus, our results provide the first evidence that development of V1–VO1 precedes that of downstream ventral regions. These findings hold important implications for understanding the origins of functional architecture in the ventral stream. First, they suggest that the early development of visual field maps V1–VO1 may be the neural scaffold that constrains the later emergence and ultimate topography of neighboring high-level visual regions^18^. Second, our findings that pRFs develop beyond V1 and after age 5 drastically extend both the length of time and expanse of visual cortex where pRF development occurs compared to what is known from research in neonate animal V1^19–21^. Future research using more complex stimuli^3–6^, participants spanning a broader age range, and longitudinal measurements can elucidate the effect of stimuli as well as the developmental trajectory of pRFs in visual cortex across childhood.

The influential eccentricity bias theory^18,30,31^ suggests that foveation on faces and words during natural viewing anchors the processing of these stimuli to regions in VTC representing the fovea. Consistent with this view, in both children and adults, the VFC in face-selective and character-selective regions is foveally biased, providing a more substantial coverage of the central than peripheral visual field. Recent intriguing resting-state connectivity data in neonate macaques^22^ show that the future site of face-selective regions shows an early functional connectivity bias with foveal eccentricity bands in V1. These data suggest that a coarse eccentricity bias in VTC may be innate or develop early during infancy via cortical connections even as pRF properties and VFC continue to develop throughout childhood. Future longitudinal research in younger participants will determine whether the over-representation of the central visual field in VTC emerges before or together with selectivity for faces or words.

Unpredicted by the eccentricity bias theory, our data show that spatial representations in these high-level regions continue to develop from childhood to adulthood. In fact, both the foveal bias and the overall VFC spanned by pRFs in face and character-selective regions increase from childhood to adulthood. These findings argue against the hypothesis that face or word selectivity develop on top of a mature foveal bias and spatial representation. The expansion of the VFC in bilateral face-selective and character-selective cortex and increase in their foveal bias may involve proliferation of dendritic arbors and synapses to support the increased pooling of information. Thus, pRF development may be associated with microstructural cortical tissue growth that has been observed in face-selective and character-selective regions^27^.

Notably, the development of pRF properties and VFC also varied by hemisphere across face-selective and character-selective regions. Character-selective regions became more foveally biased in the left-hemisphere, where previous research has demonstrated lateralization for visual word form processing and reading^42–44^. By contrast, face-selective regions became more foveally biased in the right hemisphere where face processing is thought to be lateralized^45,46^. Intriguingly, at the same time, VFC shifted away from the fovea for face-selective and character-selective regions in their non-preferred hemispheres. This pattern of development has important implications for the theory that reading and face recognition compete for foveal representations^30,31^ because it provides evidence for a competitive push-pull mechanism in which the foveal over-representation increases in one hemisphere and decreases in the other, and this process occurs in an opposing manner across hemispheres for faces and words. Additionally, the retreat of pRF coverage from the fovea in non-preferred hemispheres mirrors previous observations of development reductions in responses to non-optimal stimuli^26^.

Critically, developmental increases in both the foveal bias and VFC in face-selective and character-selective regions measured with fMRI during fixation were associated with developmental changes in fixations on faces and words measured during natural viewing outside the scanner. These data not only bridge for the first time the development of spatial processing in high-level vision and real-world viewing behavior, but also demonstrate a direct relationship between pRF properties of cortical regions and viewing behavior of complex stimuli. While our research does not inform whether behavioral changes in fixation patterns on face and word stimuli drive the development of pRFs in face-selective and character-selective regions, or if pRF development drives behavior, we note that both children and adults fixate in a way that places the VFC of face-selective and character-selective regions in an optimal location to process stimuli. In children, left pOTS-chars is less foveal, more rightward and lower-field biased compared to adults. Consequently, their fixations on words are more left and upper-field biased than adults. Likewise, in children, right pFus-faces is less foveal and more leftward shifted, consequently biasing their fixations on faces rightward compared to adults. These results, therefore, suggest a tripartite relationship between development biases in VFC in high-level regions, fixations patterns, and hemispheric lateralization. We hypothesize that this is likely an iterative and bidirectional process whereby learning optimal fixation locations (e.g., the center of a face) produces changes in the biases of visual input, altering pRFs to optimally cover regions of visual interest. Future research examining pRF development in readers of languages demanding different fixation patterns on words (e.g., Hebrew or Chinese) may explicate the interplay between viewing behavior and hemispheric lateralization.

Our findings are important not only for elucidating the development of a fundamental computation—spatial processing by RFs—in the human ventral stream and showing its relation to viewing patterns, but also for providing an innovative methodology and computational framework for investigating development of computations across cortex more broadly. As RFs are a basic hallmark of neurons in sensory cortical systems (e.g., auditory^47,48^ or somatosensory^49^ cortex), as well as complex cognitive tuning (e.g., to numerosity^50,51^) our novel approach can be applied to quantitatively examine development of cortical function throughout the brain. Likewise, our findings lay fundamental groundwork towards understanding abnormal cortical processing as well as potential maldevelopment in atypical populations, including developmental prosopagnosia^52^, dyslexia^53^, and autism^9,54^.

In summary, we find that early-developed visual field maps in the human ventral visual stream may provide a neural scaffold that shapes the organization of high-level visual regions. Furthermore, the development of pRFs in high-level visual areas involved in face and word processing is linked to changing viewing patterns on faces and words. Together, these data suggest that the spatial window through which a region of cortex processes information and our visual experience of complex stimuli changes from childhood to adulthood.

## Methods

### Participants

Twenty six neurologically typical children ages 5–12 years (mean age 8.5 ± 2.2 years, 12 females) and 26 adults ages 22–28 years old (mean age 24 ± 1.6 years, 9 females) participated in these experiments. Age ranges were chosen in children to (i) maximize a wide dynamic range of functional and structural development reported previously^23,25,26^ and (ii) maximize the success of MRI measurements without having to discard a substantial number of participants due to excessive motion in the scanner, which is a common issue with pediatric neuroimaging^55^. Because our goal was to link functional and behavioral changes, and our experiments required maintaining central fixation, we could not make measurements on younger children where acquiring such data is unfeasible. A similar range of ages was chosen in adults when most structural and functional development in VTC is thought to be near completion^56,57^. Following data quality thresholds discussed below, eight children and three adults were excluded from further analysis (18 children, 23 adults remain). Participants had normal or corrected-to-normal vision and were screened to have no prior or current psychiatric conditions. All participants could read at least simple, high-frequency words. All procedures were approved to be in accordance with the Institutional Review Board of Stanford University. Prior to the experiment, adult participants and parents provided written informed consent, and children provided written assent.

Each participant participated in several sessions completed over the course of a few months to distribute measurements and avoid fatigue. Each of the following sessions was thus performed on a different day: (i) participants under the age of 18 completed training in a mock scanner employing live feedback of head motion during the viewing of a 15-min movie. This acclimated the participants to the scanner environment and reduced motion. Participants were advanced to functional and anatomical scanning if they could lie still (less than 2.4 mm of head motion) for the duration of mock scanning. (ii) Children completed the recognition memory task with eye tracking outside the mock scanner on the same day in which they participated in training; adults completed this task after scanning was completed. (iii) All participants participated in an MRI session in which we obtained anatomical MRI brain volumes which were used to register data across sessions and obtain cortical surface reconstructions of each brain. (iv) All participants participated in an fMRI session in which we measured brain responses to stimuli of various categories (referred to as localizer experiment). (v) All participants completed an fMRI session composed of four runs of pRF mapping.

### Data acquisition

Quantitative magnetic resonance imaging acquisition: Quantitative MRI measurements are obtained from the protocols in^58^. T1 relaxation times were measured from four spoiled gradient echo (spoiled-GE) images with flip angles of 4, 10, 20, 30 (TR = 14 ms, TE = 2.4 ms) and a scan resolution of 0.8 mm × 0.8 mm × 1.0 mm. For the purposes of removing field inhomogeneities, we collected four additional spin echo inversion recovery (SEIR) scans with an echo planar imaging (EPI) read-out, a slab inversion pulse, and spectral spatial fat suppression. The SEIRs were acquired with a TR of 3.0 s, echo time set to minimum full, and 2× acceleration. The inversion times were 50, 400, 1200, and 2400 ms, and were collected at a 2.0 mm × 2.0 mm in-plane resolution and a slice thickness of 4.0 mm. An artificial T1-weighted anatomy optimized for tissue segmentation was produced for each participant from these quantitative measures which were used for surface reconstruction and visualization of retinotopic data.

Functional MRI acquisition: Data were collected on a 3-Tesla GE Discovery MR750 scanner (GE Medical Systems) at the Center for Cognitive Neurobiological Imaging at Stanford University using a phase-array 32-channel head coil. Functional data for the category localizer were collected with a simultaneous multi-slice EPI sequence with a multiplexing factor^59^ of 3 to acquire near whole-brain (48 slices) volumes at TR = 1 s, TE = 30 ms. Data were acquired at a resolution of 2.4 mm isotropic voxels with one-shot T2*-sensitive gradient echo sequence with slices aligned parallel to the parieto-occipital sulcus. Functional data for retinotopic mapping were of similar resolution and orientation but collected on a 16-channel head coil, TR = 2 s, acceleration factor of 2, 28 slices.

fMRI category localizer experiment: The purpose of this experiment was to identify those voxels whose neural response preferred either faces or words in order to localize face-selective and character-selective cortex as functional regions of interest. During scanning, participants completed 3 runs, each 318 s long, of an experiment presenting participants with stimuli from five categories each with two subcategories (faces: child, adult; bodies: whole, limbs; places: corridors, houses; objects: cars, guitars; characters: words, numbers) as described previously^24,27,60^. Images of a category were presented in 4 s miniblocks at a rate of 2 Hz and did not repeat across miniblocks or runs. Each category was shown eight times in a run in counterbalanced order interleaved with blanks. Participants fixated on a central dot and performed an oddball detection task of phase scrambled images.

pRF mapping experiment: The purpose of this experiment was to model in every voxel the region of the visual field that is capable of eliciting a response from that voxel, namely its RF. Participants completed 4 runs of an experiment in which participants fixated on a central stimulus and were required to indicate via a button-press when the central stimulus changed color. Black and white checkerboard bars (width = 2° of visual angle, length = 14°) were swept across the screen during each run which lasted 3 min and 24 s. Bars swept the visual field in eight different configurations in each run (four orientations: 0°, 45°, 90°, 135°, each orientation was swept in two directions that were orthogonal to the bar). Same as^17^. We used checkerboard stimuli as they are the most ubiquitous stimuli that is used for pRF mapping, activates the majority of the ventral stream, and does not require cognitive processing that may differ across age-groups.

Eye-tracking and fixation task performance were collected on a subset of children and adults. Fixation performance on participants was tracked with the Eyelink software (http://www.sr-research.com/). Blinks, labeled by the Eyelink software, were removed from the timecourse data of the recorded eye by scrubbing with a 100 ms window on either end of the blink. Fixation data was then plotted for each participant. Only participants that made fewer than three saccades (2° in size) during a mapping run were included for analysis. Due to the scanner environment, size of participants’ head, and time constraints, not all participants could be eye-tracked during pRF mapping (eye tracking data was obtained for 25 children and six adults). Fixation task performance was also only collected on a subset (eight children, seven adults) of participants due to button box malfunction. All participants, however, were trained on proper fixation technique during the recognition memory task (see Behavioral data and analysis below), and all participants included in the analysis that underwent eye-tracking in the scanner fixated successfully, with no difference between age-groups. As a reminder, we also observe no difference in pRF properties or pRF model performance in V1 between children and adults, further suggesting proper fixation performance, as improper fixation significantly impacts pRF size estimates^61^.

### Data analysis

Anatomical data analysis: Both the spoiled-GE and the SEIR scans were processed using the mrQ software package in MATLAB to produce T1-weighted maps^58^. The mrQ analysis pipeline corrects for RF coil bias using SERI-EPI scans, producing accurate proton density (PD) and T1 fits across the brain. The full analysis pipeline and its published description can be found at (https://github.com/mezera/mrQ). An artificial T1-weighted anatomy was produced for each participant from these quantitative measures which were used for surface reconstruction and visualization of retinotopic data. Anatomical images for each participant were segmented through FreeSurfer (https://surfer.nmr.mgh.harvard.edu/), the resultant tissue segmentation was hand-corrected for classification errors. Functional data were restricted to the cortical ribbon by growing a 3-voxel thick (1 mm isotropic voxels) ribbon from the gray-white matter boundary.

fMRI data analysis: Data were processed and analyzed in MATLAB using mrVista software (http://github.com/vistalab) as in previous publications^24,27^. Functional data were aligned to the artificial T1-weighted volume. Functional data were unsmoothed, always analyzed within the individual participant native brain anatomy space, and were restricted to the cortical ribbon.

Functional data were motion corrected both within and between scans. Any participants who moved more than 2 voxels within a scan were either excluded from data analysis or invited back for another session, such that children and adults were matched for data quality as shown in Supplementary Fig 2C. There was no significant difference in motion during scanning between groups (see Results paragraph 1). To ensure there were no group differences between children and adults resulting from differences in data quality, age-groups were matched for the mean percentage variance explained of the pRF model across voxels in V1, resulting in no significant difference in explained variance across all visual field maps (*F*_(1,185)_ = 0.59, n.s., 2-way ANOVA with factors of ROI and age).

Definition of V1–VO1: Maps of pRF phase and eccentricity were projected onto an inflated cortical surface reconstruction for each participant (Supplementary Figs. 3–6). Borders between retinotopic maps were drawn on the cortical surface. The boundary was defined as the center of polar angle reversals occurring at the vertical or horizontal meridian^16,62,63^ for V1, V2, and V3. hV4 and VO1 were defined following^16,62–64^. As a guiding rule to ensure consistent map definition across participants, we delineated visual field representations using polar angle reversals near the following anatomical landmarks: V1/V2 ventrally on the superior portion of the lingual gyrus and dorsally on the inferior portion of the cuneus, V2/V3 ventrally near the lingual sulcus and dorsally near the superior portion of the cuneus, V3/hV4 on the posterior transverse collateral sulcus (ptCoS), hV4/VO1 on the ptCoS to medial fusiform/CoS. Unlike the maps surrounding the confluent fovea (V1–hV4) which share an eccentricity representation, and whose borders are defined by reversals in polar angle preference^60^, hV4 and VO1 share a polar angle representation, and their boundary is defined by a reversal in the eccentricity preference as illustrated in^63^. In some cases, the lower vertical meridian of hV4 may be hard to image due to an MR artifact arising from the transverse sinus (referred to as the “venous eclipse”^65^). However, the sinus artifact tends to affect BOLD signals on the inferior occipital gyrus (IOG), and in our data, does not interfere with the BOLD signals in the ptCoS, which is the location of the eccentricity map reversal specifying the boundary between hV4 and VO1. V1, hV4^63,64^, and VO1^63,64^ were drawn as hemifields representing the contralateral visual field. V2 and V3 were drawn as quarterfields separated by V1, and were later combined to produce a hemifield representation. Individual maps were drawn by JG and independently checked by VN and KGS.

We also used an independent anatomical definition of V1^66^ to assess if using an ROI defined independently from the data had any impact of pRF quantification or developmental findings. Using cortex-based alignment in FreeSurfer^67^, we transformed V1 defined anatomically on the FreeSurfer average brain (generated from 39 independent adults) into each individual participant’s brain and repeated analyses presented in the main text. Results shown in Supplementary Fig. 8 replicate the main results shown in Fig. 2, showing no development of pRF properties or VFC in V1 after age 5.

Definition of face-selective and character-selective functional regions of interest: Statistical contrasts of faces or characters > all other stimuli were thresholded at *t*-values > 3, voxel level, for all participants, as in our previous work^24,27,60^. Face-selective voxels that responded more strongly to faces than other stimuli and were located in the posterior lateral fusiform gyrus were defined as pFus-faces/FFA1. Character-selective voxels that responded more strongly to pseudowords and number strings than all other stimuli that were located on the posterior occipitotemporal sulcus lateral to pFus-faces were defined as pOTS-chars as in^60^. This region is also defined elsewhere as VWFA1^60,68^ using real word stimuli. Given that our region (pOTS-chars) occupies the same anatomical location as VWFA1, character-selective and word-selective are treated as synonymous in this study. See Supplementary Fig. 9 for example ROIs.

Estimating pRFs: After functional data were transformed to the whole brain anatomy and restricted to the cortical ribbon, a pRF model was fit in each voxel^17^. For each voxel, a 2-dimensional Gaussian receptive field is modeled, having a center described by x and y coordinates and a sigma describing the width, and a parameter, g, describing its gain. An additional variable is fit for each voxel describing a compressive summation factor of the product of the stimulus and the Gaussian receptive field to better describe nonlinear summation properties of cortical responses as one ascends the visual hierarchy^35^. A candidate timecourse is produced from this pRF by convolving an HRF with the product of the stimulus movie and the pRF. The variables *x*, *y*, and sigma are swept until the variance explained of the voxel’s timecourse is maximized by the pRF model. Voxels were only included for subsequent analysis if the variance explained by the pRF model was greater than 5%. Additionally, to ensure the most accurate pRF fits, voxels whose pRF centers were outside the stimulus field (>7° radial eccentricity) or whose sigma was assigned the model’s minimum/floor value (0.21°) were excluded from further analysis.

Data include all voxels in which the pRF model explained at least 5% of their variance. To estimate if this is an adequate threshold, we evaluated the distribution of the variance-explained in voxels in primary auditory cortex. To do so, we projected the FreeSurfer ROI in the superior temporal gyrus ROI that encompasses A1 from the FreeSurfer average cortical surface into each participant’s native cortical surface. In each voxel of this ROI we evaluated the variance-explained of the pRF model. Across all participants, the vast majority of voxels in this ROI had a variance-explained of zero, and the variance-explained in 94.4% of the voxels was less than 5%. This analysis demonstrates the sufficiency of this threshold to exclude non retinotopic voxels. With this threshold, we also find a similar number of voxels in retinotopic areas of children and adults, which enables a fair comparison across age-groups (see Supplementary Fig. 2).

pRF size vs. eccentricity fits: To evaluate the relationship between a pRF’s size and its eccentricity shown in Fig. 2, voxels within an individual’s ROI were entered into a linear regression in which each voxel’s contribution was weighted by the variance explained of the pRF model. Only voxels with greater than 5% variance explained were included. The line-of-best fit was derived in each participant for each ROI, and then the slope and intercept of this line was averaged across participants of each age group.

VFC analyses: To calculate the VFC for a given ROI and participant, all voxels in an ROI that contain pRFs with >5% variance explained by the model are included and modeled as a Gaussian with a peak normalized to 1. The VFC is produced at each point by averaging the value across pRFs that cover that point, and then normalizing by the maximum coverage value in that participant. We also implemented a bootstrapping procedure that draws with replacement n-voxels from a participant’s ROI of size n, and produces an average VFC from 50 iterations to reduce the effect of outlier voxels. The average VFC from this bootstrapping approach is the VFC used for a given participant’s ROI. To produce the average VFC of participants in each age group (Figs. 2 and 4), the VFC is averaged across participants of an age group. For the VFC of the visual field maps shown in Fig. 2 we first flipped for each participant the VFC of right hemisphere map over the vertical meridian and averaged with left hemisphere VFC before averaging across participants. To measure the extent of the VFC for face-selective and character-selective regions (Fig. 4f), we estimated the bilateral VFC for pFus-faces and pOTS-chars in each participant. pRF coverage density was binarized in each participant’s ROI by setting any coverage less than 0.01 to zero (non-zero coverage assigned a value of 1) and the proportion of the visual field covered was multiplied by the total area stimulated by the sweeping bar stimulus (*πr*^2^, *r* = 7°), resulting in the square degrees of visual angle covered by an individual’s ROI. We then averaged this across participants in a group.

Center-of-mass distance from fixation: To quantify the foveal bias observed in face-selective and character-selective regions, we computed the center-of-mass (CoM) distance of the VFC of each region from the center of the visual field (Fig. 4). This was derived by multiplying each coordinate by the normalized coverage density to obtain the center of VFC in a given region within children or adults. This measure was then jackknifed, repeated n times leaving out *n*−1 participants on each fold, to produce the bars of standard error.

### Behavioral data and analysis

Participants completed a recognition memory behavioral experiment while being eye-tracked with an Eyelink 1000 eyetracker (www.sr-research.com) in our eye tracking lab. The goal of the experiment was to measure fixation patterns during free viewing of face and word stimuli outside the scanner, participants were seated, head-fixed using a chin rest and positioned 54 cm from a monitor and told to freely view stimuli. The experiment had three parts: (1) Encoding: participants viewed images from five categories (child/adult faces, indoor/outdoor scenes, car/guitar objects, word/number characters, whole bodies/limbs) and performed a 1-back task, indicating when two consecutive images were identical. Visual stimuli subtended 4°–7° of visual angle, presented centrally within a 9° square (see Fig. 5 for examples). (2) Fixation: participants were instructed to fixate on a central dot while viewing a rotating checkerboard. This part was ~4 min long and served to train the participants to fixate during subsequent pRF mapping experiments. (3) Recognition memory: immediately followed the fixation training. Here, participants were presented with a surprise recognition task in which images appeared on the screen and for each image they were asked to indicate if it was previously seen during the Encoding phase or if was a new image. This part was self-paced and the images appeared on the screen until participants made a decision. Between stimuli (inter-trial interval of 1 s) there was a fixation dot to orient the participant to the center of the screen. This dot disappeared when the stimulus appeared. Images were randomly presented and were slightly jittered in their position. There were 16 images per category (faces, words), each presented once. We report all fixations made during the viewing of a given stimulus (not just the first), as quantified in Fig. 5 and Supplementary Fig. 12. We only use fixations from the recognition phase of the experiment (we observed no significant differences from fixations made during encoding). Because the stimuli varied in position and size, we defined for each stimulus the AFZ (described below) and quantified how children varied from this zone for each image, thus avoiding any potential misalignment issues between stimuli.

Eye movement analysis: After removing timepoints during which participants blinked, data presented in Fig. 5 were analyzed in the following way: Fixation patterns were plotted in a 2-dimensional matrix (768 × 1024 pixel grid, equal in size to the stimulus presentation screen) and smoothed with a small Gaussian filter (sigma = 18.75 pixels) for the purpose of averaging data across participants. Fixation density was normalized by the maximum in each participant, and then averaged for a given stimulus across all participants of an age group. The adult average fixation density was thresholded at 70% overlap for each stimulus and defined as the adult fixation zone (AFZ). The ratio of individual fixations made inside vs. outside this this AFZ was calculated for each child participant and image, and then averaged across participants and stimuli of a given class (e.g., faces). The ratio was defined as (fixation time outside AFZ)/(total fixation time). A value of 1 indicates that all fixations occurred outside the AFZ, and value of 0 indicates that all fixations were within the AFZ. We then calculated if children fixated outside the AFZ significantly higher than chance, chance here being that 30% of fixations would occur outside the AFZ (as it was defined in adults as the 70% overlap contour). Because stimuli were slightly jittered, the AFZ analysis allows us to determine what adult-like fixation patterns should look like for each individual stimulus regardless of that stimulus’ position or orientation. By comparing individual children to the AFZ within each individual image, we can avoid misalignment issues between jittered stimuli, and this test allows us to directly test how children deviate from adult-like behavior without making any assumptions about the data.

Fixation bias vector analysis: The average fixation density for each face and word stimulus from the visual recognition test was calculated separately for children and adults. We first calculated for each image the center-of-mass of the distribution of adult fixations, similarly to the adult fixation zone analysis discussed above, finding the center of the zone where 70% of adults fixated. We then calculated the center of mass of child fixations. From this center, a vector was produced pointing towards the center of fixation density on the same stimulus in children. Bias in child fixation vectors (Fig. 5c) was quantified using a *t*-test to determine if vectors for a given stimulus category significantly deviated away from the quadrant that contained the VFC (for example, the coverage of right pFus-faces in the lower left quadrant) which we term the null quadrant. This procedure tested the hypothesis that children fixate in an optimal manner (e.g., they do not fixate in such a way that would move their limited coverage away from the informative region in the stimulus). It was assumed that 25% of randomly distributed vectors would have angles within the null quadrant (if randomly distributed, 25% of vectors should lie in quadrant spanning a quarter of the visual field). Thus, for each vector, we calculated its angular distance from the null quadrant (for example, a vector pointing 15 degrees into the upper right quadrant is, clockwise, 105 degrees from the lower-left quadrant). This was repeated for each vector, and for this population *t*-tests were performed to assess if resulting bias vector thetas significantly deviated from this null.

### Statistical analyses

*N*-way ANOVAs were run for data presented in Fig. 2 with appropriate grouping variables and revealed no main effects or interactions, and thus no *t*-tests or KS-tests were performed. For Fig. 3, *N*-way ANOVAs were run for pRF size and eccentricity treating ROI, hemisphere, and age-groups as separate variables. For all statistical tests, we report any significant main effects or interactions. Data going into ANOVAs was tested for normality assumptions using a Lilliefors test, and all data met or were very close to normal. ANOVAs are robust against modest deviations from normality, and no data populations have any gross violation of normality. All *t*-tests or KS-tests conducted were two-tailed. To test if any of our effects were correlated with age, we calculated the Pearson correlation coefficient between the neural data and age on data underlying Figs. 2 and 4. These values are reported in the text with the number of participants going into each correlation. None of these correlations were significant. Bootstrapping methods were used to produce VFC plots in Figs. 2 and 4 to ensure robustness of fits and downweight outlier voxels; this bootstrapping method is described in the section VFC analyses, above. For Fig. 4a–d, a two dimensional, 2-sample Kolmorogov-Smirnov test^69^, which is a nonparametric test comparing two continuous distributions simultaneously along two dimensions, was run on each ROI to test if the VFC was different across age-groups. All errorbars in the main and supplementary figures represent standard error of the mean across participants.

### Data availability

All code relevant to data analysis for the main findings (Figs. 1–5) is available on github.com/VPNL. Any source data relevant to these analyses will also be made available upon request. The majority of the code used in this study was derived from scripts and functions available through the open-source vistasoft code library: https://github.com/vistalab/vistasoft.

## Electronic supplementary material

Supplementary Information
