# Supplementary material for: Development differentially sculpts receptive fields across early and high-level human visual cortex
Source: Nat Commun. 2018 Feb 23;9:788. doi: 10.1038/s41467-018-03166-3 (PMC5824941; doi:10.1038/s41467-018-03166-3)
Supplement: Supplementary file 1 — Supplementary Information [file 41467_2018_3166_MOESM1_ESM.pdf]

Supplementary Figures

Development differentially sculpts receptive across early and high-level human visual cortex

Supplementary Figures 1-12

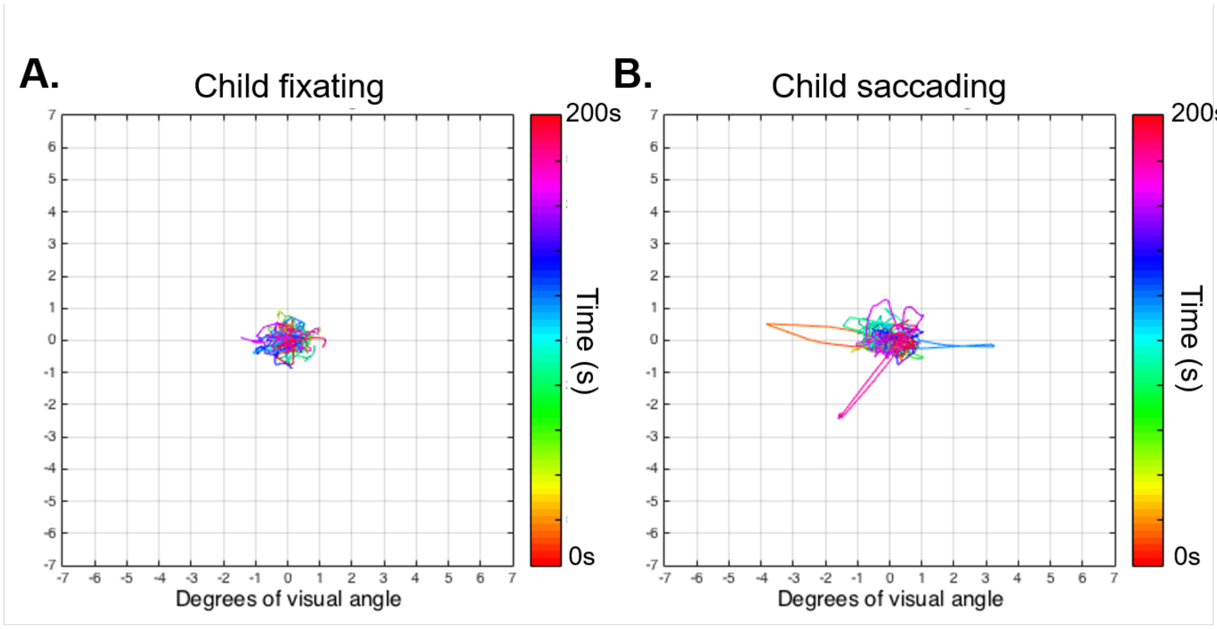

C.

	Mean # of Saccades	Mean Accuracy
**Ages 5-12**	1.48 (± 0.44)	99.67% (± 0.23)
**Ages 22-28**	0.48 (± 0.28)	98.43% (± 0.74)

Supplementary Figure 1: Fixation and behavioral performance during retinotopic scanning. Fixation patterns from example participants either fixating (A) or (B) making minor saccades. The fixation path is color coded according to time (seconds) during the retinotopic mapping. Small deviations from the center are likely microsaccades and pupil-tracking noise from the scanner environment. (C) Behavioral performance during pRF mapping. Numbers indicate mean and standard deviation.

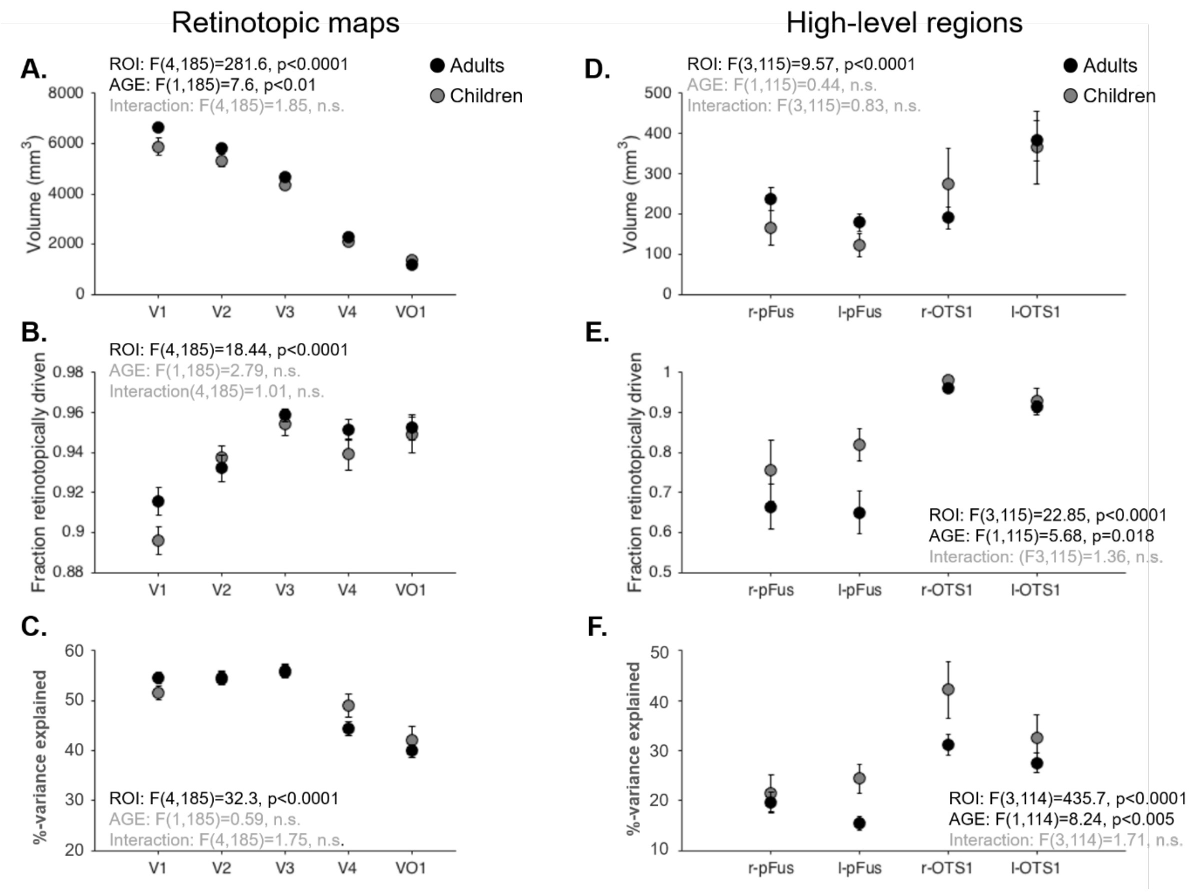

Supplementary Figure 2: Summary characteristics of retinotopic maps V1-VO1 and regions of VTC. (A) Volume in cubic millimeters is reported from visual field maps V1 through VO1. Error bars represent standard error. Reported volume measurements are the volume of voxels within the map that survive variance-explained thresholding. Children are light gray, adults black. Participants included are matched for variance explained in V1. Participant numbers are the same as those reported in Figures 2 and 4. **(B)** The proportion of an ROI that is retinotopically driven above the 5% variance-explained threshold. Children are gray, adults black. **(C)** The mean percentage of variance explained across ROIs in children (gray) and adults (black) after variance-explained thresholding. **(D-F)** Same as A-C but for face- and word-selective regions. The r- and l- denote right and left hemisphere. ANOVAs for each were run with grouping variables of ROI and age-group. Main factors are reported, followed by the interaction.

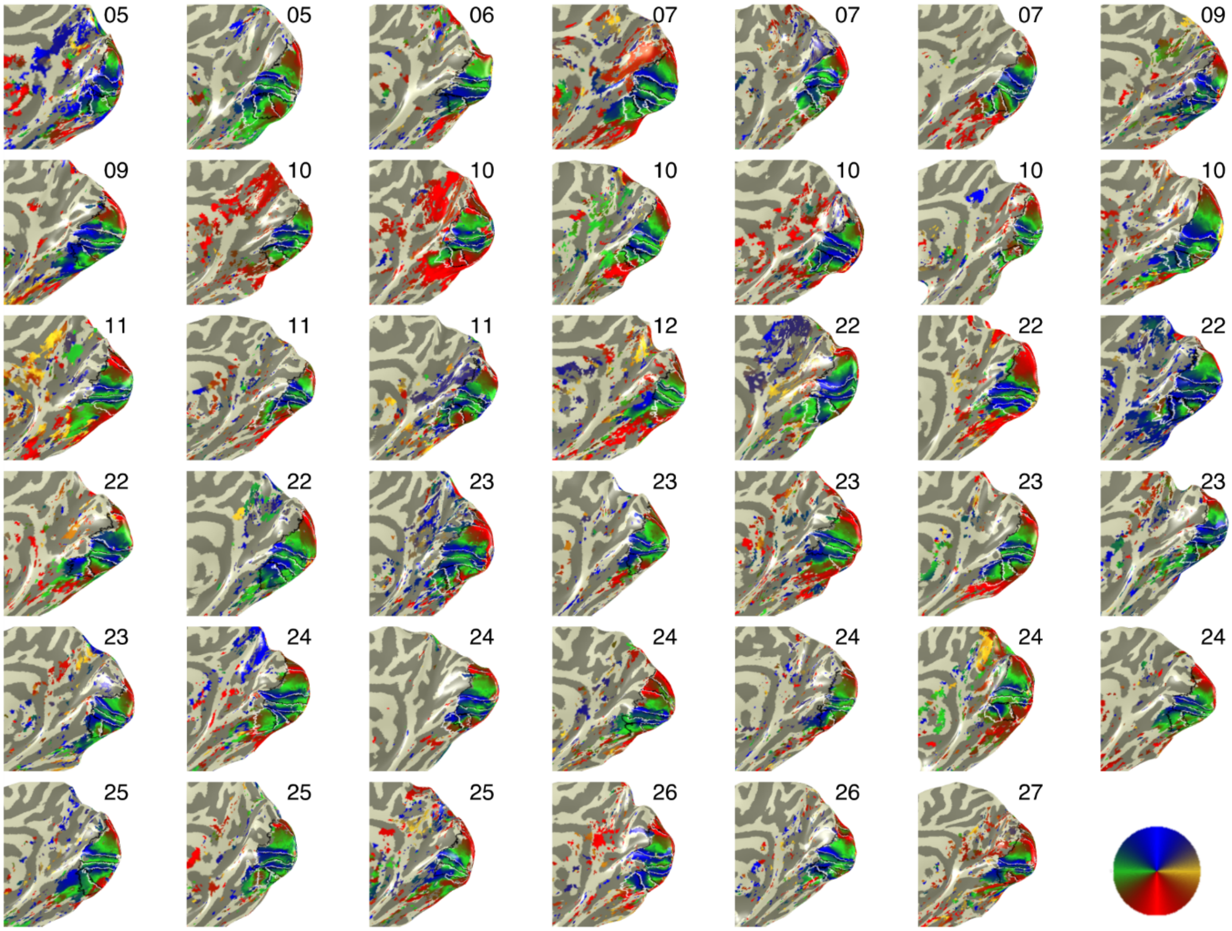

Supplementary Figure 3: Polar angle maps of the right hemisphere occipital and temporal lobes for all participants. Voxels are thresholded at 5% variance explained. All maps that we defined are presented, including V1, V2, V3, hV4, VO1. Not all maps could be delineated in each participant. Numbers indicate the age of the participant. Color wheel*:* polar angle color coding.

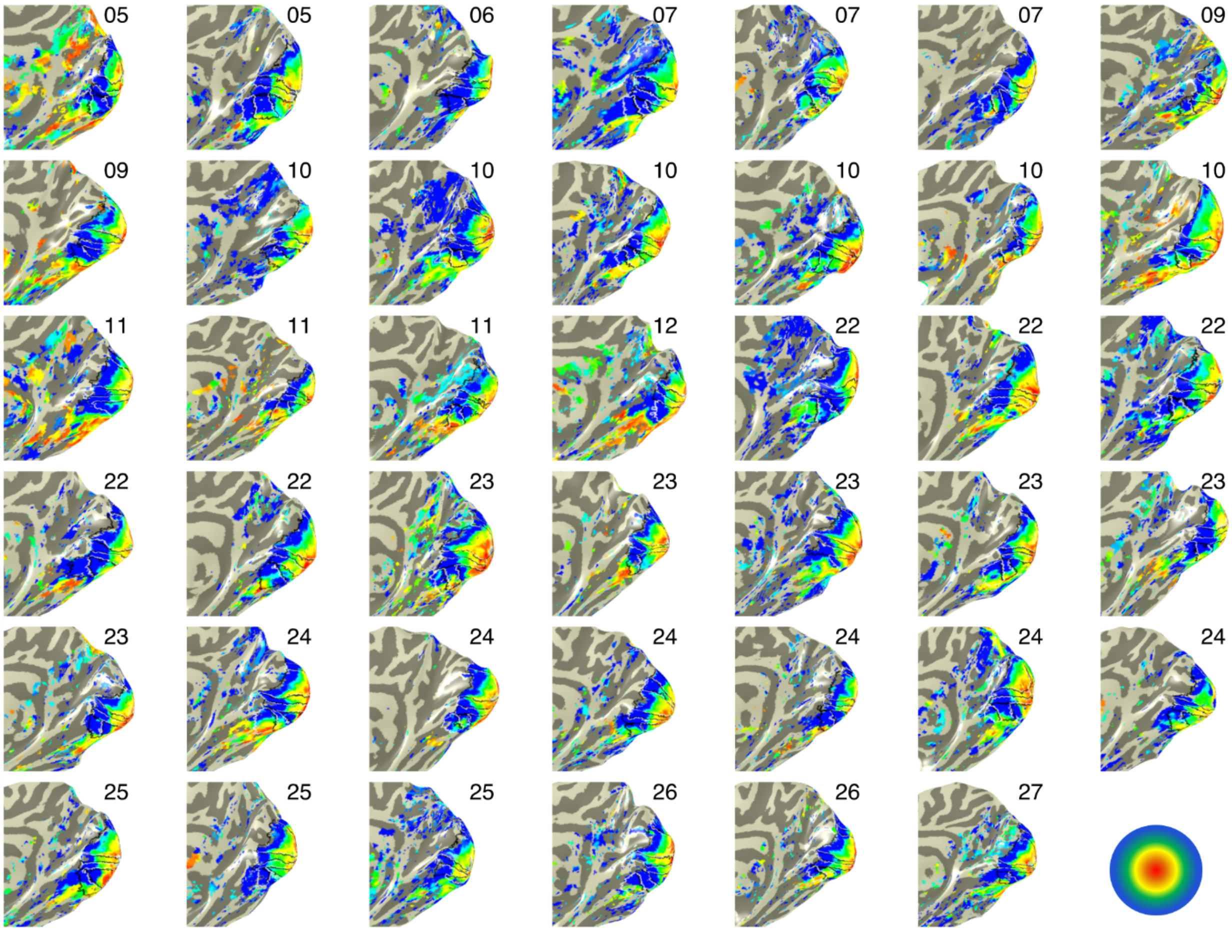

Supplementary Figure 4: Eccentricity maps of the right hemisphere occipital and temporal lobes for all participants. Voxels are thresholded at 5% variance explained. All maps that we defined are presented, including V1, V2, V3, hV4, VO1. Not all maps could be delineated in each participant. Numbers indicate the age of the participant. Color wheel*:* eccentricity color coding.

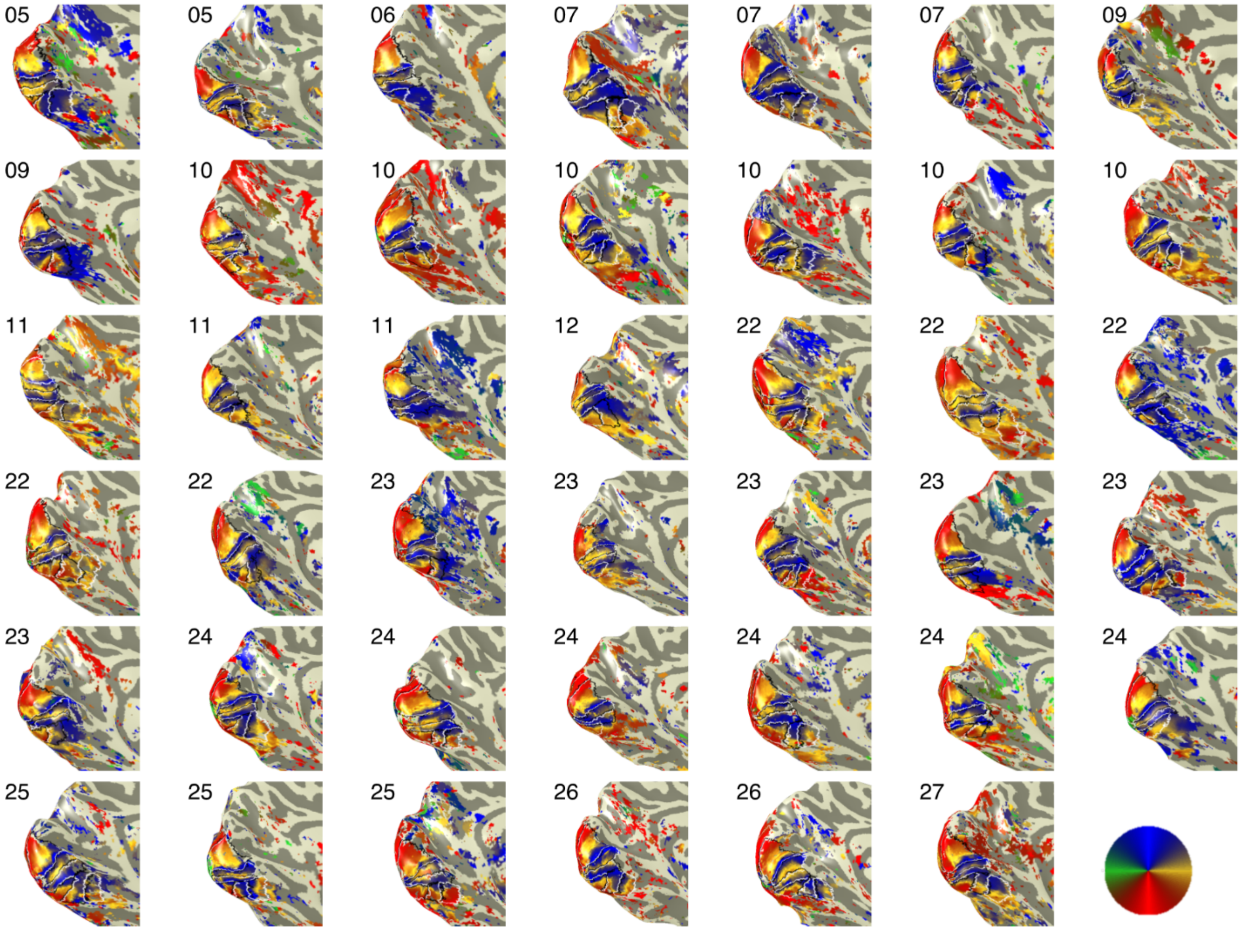

Supplementary Figure 5: Polar angle maps of the left hemisphere occipital and temporal lobes for all participants. Voxels are thresholded at 5% variance explained. All maps that we defined are presented, including V1, V2, V3, hV4, VO1. Not all maps could be delineated in each participant. Numbers indicate the age of the participant. Color wheel: polar angle color coding.

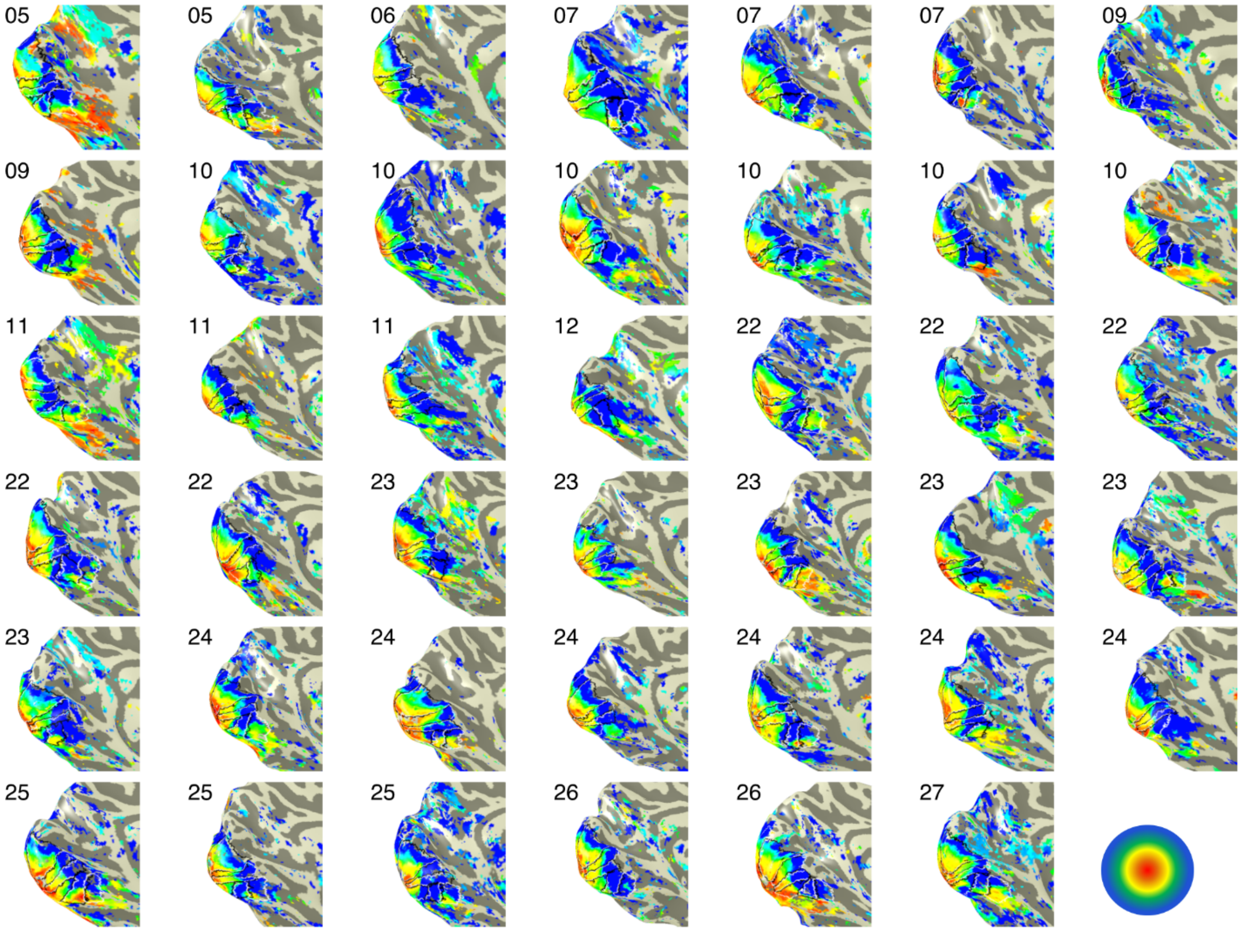

Supplementary Figure 6: Eccentricity maps of the left hemisphere occipital and temporal lobes for all participants. Voxels are thresholded at 5% variance explained. All maps that we defined are presented, including V1, V2, V3, hV4, VO1. Not all maps could be delineated in each participant. Numbers indicate the age of the participant. Color wheel: eccentricity color coding.

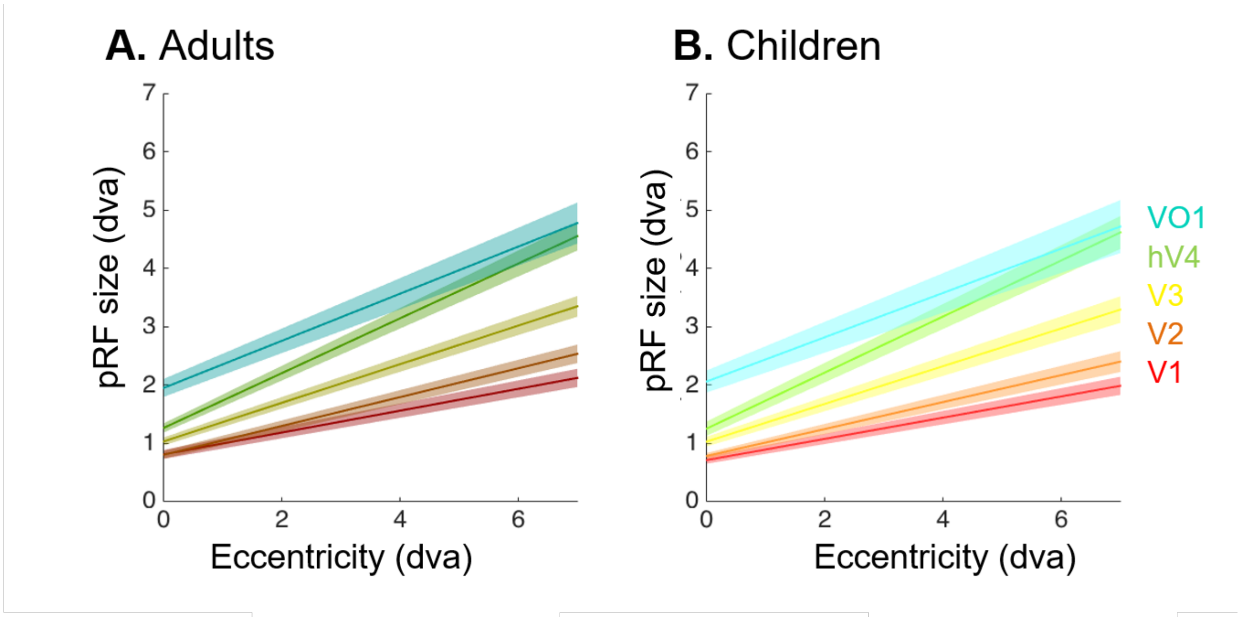

Supplementary Figure 7: pRF size versus eccentricity fits in children and adults are similar. The line of best fit (solid line) and the standard error (shaded region) illustrates the relationship between pRF eccentricity and size in units of degrees of visual angle (dva). **(A)** Fits for V1 through VO1 are plotted for 23 adults. **(B)** Same as A but for 18 children.

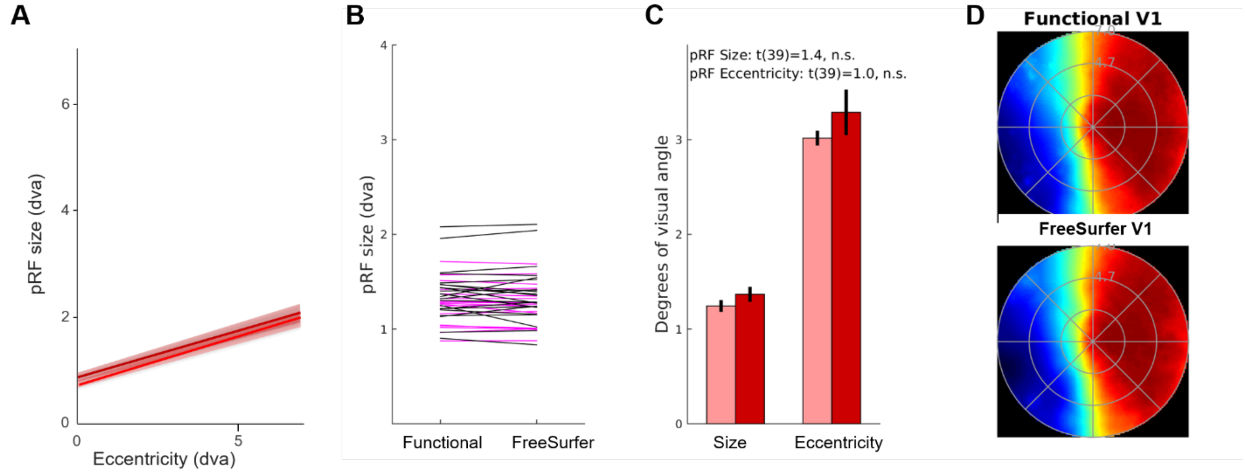

Supplementary Figure 8: Cross-validation, no development of pRF properties and visual field coverage in an independently defined V1. V1 was defined using an independent approach in which we used cortex-based alignment in FreeSurfer to transform the average V1 defined on the FreeSurfer average brain into each individual's cortical surface. (A) Linear fits relating pRF size and eccentricity within FreeSurfer V1 for children (light red) and adults (dark red). Solid lines are mean line-of-best-fit in each group, shaded regions are standard error. There is no significant difference between the intercept or slope of these fits between groups, as reported for functionally-defined V1 in the main text. (B) Individual participant lines showing that the mean pRF size does not change if V1 is defined functionally or anatomically in FreeSurfer. Children are shown in magenta, adults in black. (C) Quantification of mean pRF size (left two bar graphs) and eccentricity (right two bar graphs) in children and adults (light and dark red) within FreeSurfer V1. As reported in the main text for functionally-defined V1, there remains no significant difference between pRF properties. (D) Average visual field coverage in functionally-defined V1 (top) and FreeSurfer V1 (bottom) is identical in child participants.

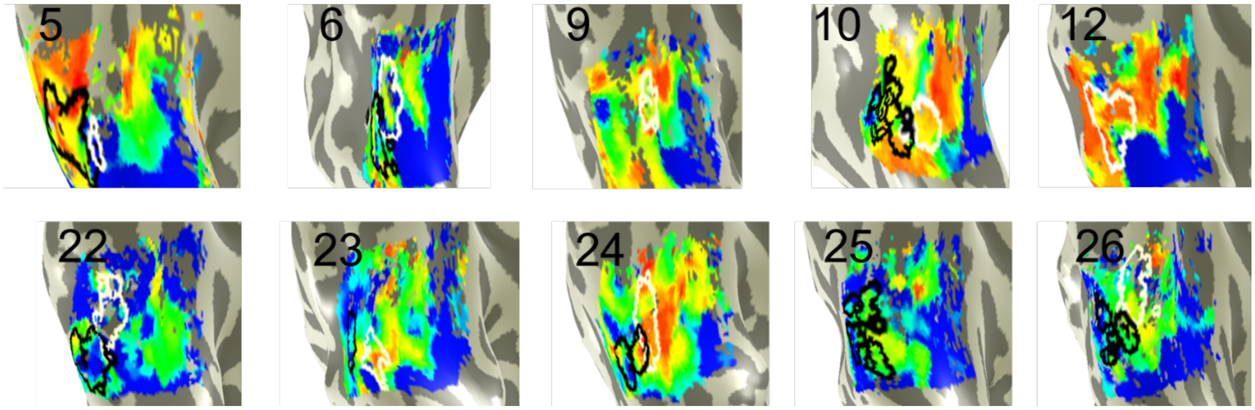

Supplementary Figure 9: Example category-selective regions in the eccentricity representation of ventral temporal cortex. Face- (white outlines) and word-selective regions (black outlines) are show in 5 example children (top row) and 5 adults (bottom) overlaid on each participant's eccentricity map. The age of each participant is present in the upper left of each brain surface. Eccentricity has been masked to the ventral temporal cortex (bound by the collateral sulcus, occipitotemporal sulcus, posterior-transverse collateral sulcus, and anterior fusiform gyrus). The 9yo, 12yo, and 25yo represent participants in which we could not define one of the regions of interest. The number of participants in which an ROI could be defined is shown in the coverage plots of Fig 4.

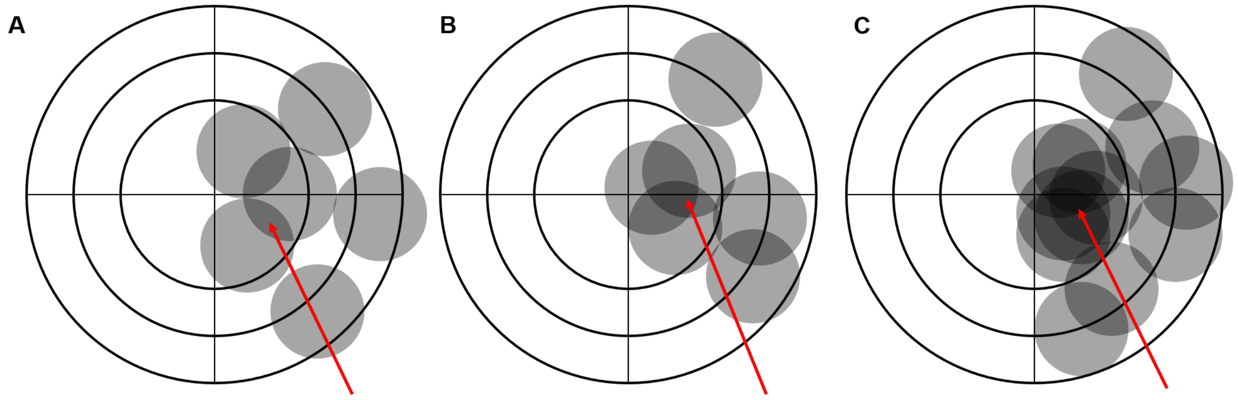

A: Here, there are three pRFs with centers at <2°, and 3 centers out in the periphery. Here, there is a foveal bias (red arrow points to the coverage maximum with 2 overlapping RFs).

B: Here, the same number of receptive fields with the same mean eccentricity but with more consistent overlap in the fovea can lead to an increase in coverage of the fovea (max coverage shown by red arrow is now covered by 3 pRFs).

C: Here, the mean pRF eccentricity is identical to the cases on the left (6 pRF centers at <2°, and 6 out in the periphery). However, because the pRFs more consistently overlap a given region in the fovea, you can see that the foveal coverage bias has increased, as the same region in the fovea is now being covered twice as densely as before.

Supplementary Figure 10: Visual field coverage and foveal bias depends on three factors: pRF center, pRF size and pRF scatter. The scenarios above illustrate situations in which the foveal bias increases from A->B or A->C without a change in mean pRF eccentricity or size. That is, scatter alone can affect the center of mass of the visual field coverage**.**

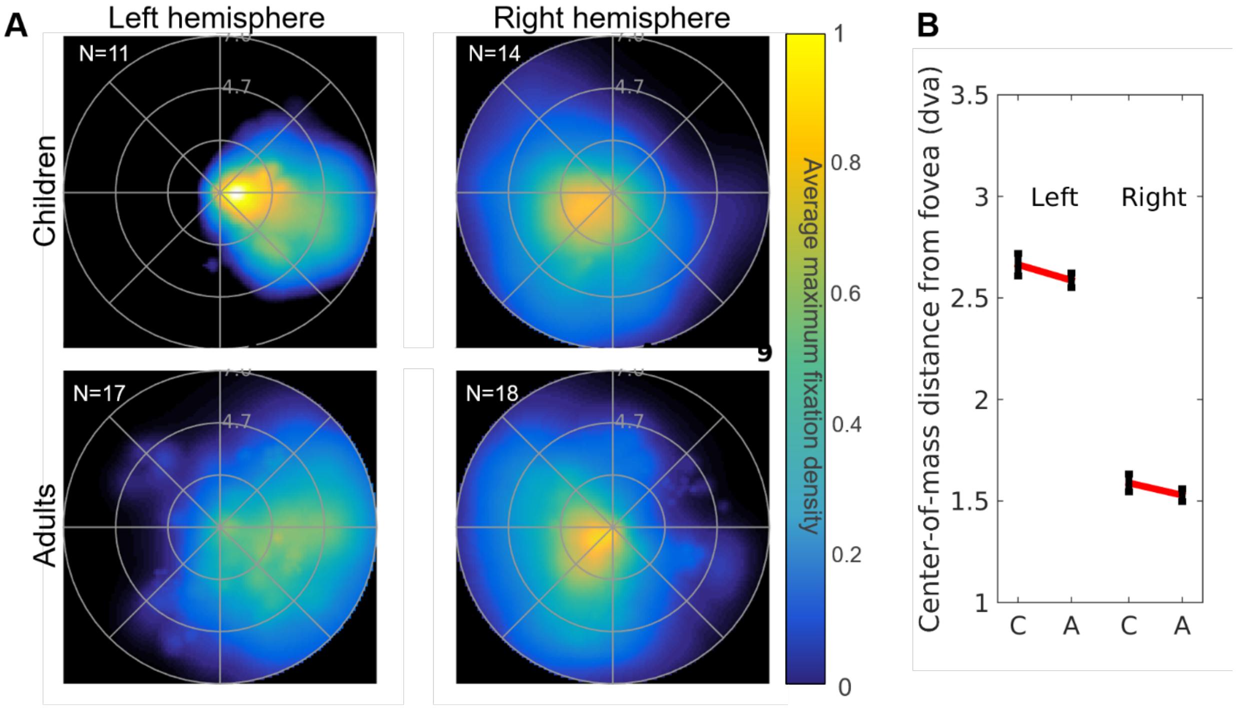

Supplementary Figure 11: Visual field coverage in matched size pFus-faces. Left and right pFus-faces in children were dilated to match the average adult ROI size. (A) Average visual field coverage in children (top) and adults (bottom) In the left hemisphere the visual field coverage became less foveal from childhood to adulthood, and in the right hemisphere the visual field coverage became more foveal. (B) The center of mass (CoM) of the visual field coverage. Error bars: SEM. Results are similar to main data presented in Figure 4, suggesting ROI size is not a factor driving the effect of pRF coverage development.

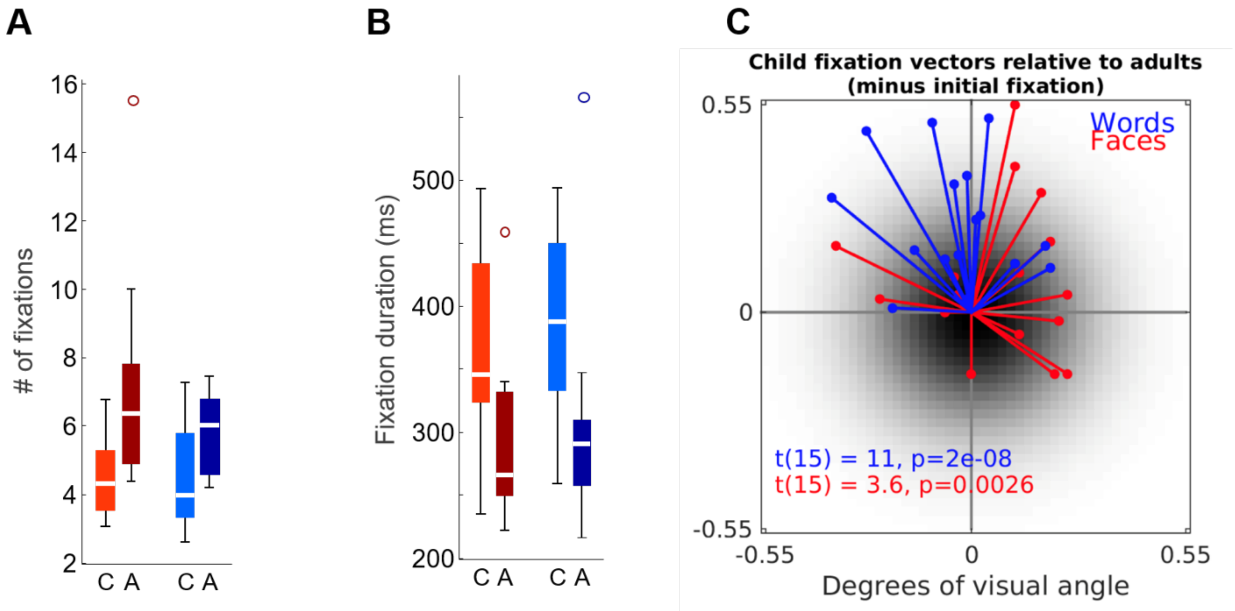

Supplementary Figure 12: Fixation number and duration on face and pseudoword stimuli in children and adults during the recognition task. (A) The number of fixations on faces and pseudowords in children (light colors) and adults (darker color) during the recognition task. Adults make significantly more fixations than children ($t(21)=2.7$, $p<0.02$), but they are clustered near the center of the stimulus (**Fig 5**). (B) Fixation duration on faces and pseudowords in children (light colors) and adults (dark colors) during the recognition task. Adults' fixation durations are significantly shorter compared to those of children ($t(21)=2.4$, $p<0.03$). White line: median; Box: 25th and 75th percentiles; Whiskers: range; Circles: outliers; C: children; A: adults; red: unfamiliar faces; blue: pseudowords. **(C)** Analysis of fixation bias on each image, excluding the initial 10% of fixation duration. Vectors describing the bias in child fixation densities for face (red) and pseudoword (blue) stimuli relative to the center of adult fixation densities for each stimulus. Each vector is the bias for a particular stimulus. Black Gaussian center represents the centrally-biased adult fixation densities. T-tests evaluate if vectors are radially different across age groups.
